# Molecular surveillance for anti-malarial drug resistance and genetic diversity of *Plasmodium falciparum* after chloroquine and sulfadoxine-pyrimethamine withdrawal in Quibdo, Colombia, 2018

**DOI:** 10.1186/s12936-022-04328-x

**Published:** 2022-10-28

**Authors:** Angela Patricia Guerra, Mario Javier Olivera, Liliana Jazmín Cortés, Stella M. Chenet, Alexandre Macedo de Oliveira, Naomi W. Lucchi

**Affiliations:** 1 Grupo de Parasitología, Instituto Nacional de Salud, Bogotá, Colombia; 2 Instituto de Investigaciones en Ciencias Biomédicas, Universidad Ricardo Palma, Lima, Perú; 3 Malaria Branch, Division of Parasitic Diseases and Malaria, Center for Global Health, Centers for Disease Control and Prevention, GA Atlanta, USA

**Keywords:** Colombia, Molecular markers, Chloroquine, Sulfadoxine-pyrimethamine, Neutral microsatellites, *Plasmodium falciparum*

## Abstract

**Background:**

Resistance to anti-malarial drugs is associated with polymorphisms in target genes and surveillance for these molecular markers is important to detect the emergence of mutations associated with drug resistance and signal recovering sensitivity to anti-malarials previously used.

**Methods:**

The presence of polymorphisms in genes associated with *Plasmodium falciparum* resistance to chloroquine and sulfadoxine-pyrimethamine was evaluated by Sanger sequencing, in 85 *P. falciparum* day of enrollment samples from a therapeutic efficacy study of artemether–lumefantrine conducted in 2018–2019 in Quibdo, Colombia. Samples were genotyped to assess mutations in *pfcrt* (codons 72–76), *pfdhfr* (codons 51, 59, 108, and 164), and *pfdhps* genes (codons 436, 437, 540, and 581). Further, the genetic diversity of infections using seven neutral microsatellites (NMSs) (C2M34, C3M69, Poly α, TA1, TA109, 2490, and PfPK2) was assessed.

**Results:**

All isolates carried mutant alleles for *pfcrt (*K76**T** and N75**E)***,* and for *pfdhfr* (N51**I** and S108**N)**, while for *pfdhps*, mutations were observed only for codon A437**G** (32/73, 43.8%). Fifty samples (58.8%) showed a complete neutral microsatellites (NMS) profile. The low mean number of alleles (2 ± 0.57) per locus and mean expected heterozygosity (0.17 ± 0.03) showed a reduced genetic diversity. NMS multilocus genotypes (MMG) were built and nine MMG were identified.

**Conclusions:**

Overall, these findings confirm the fixation of chloroquine and pyrimethamine-resistant alleles already described in the literature, implying that these drugs are not currently appropriate for use in Colombia. In contrast, mutations in the *pfdhps* gene were only observed at codon 437, an indication that full resistance to sulfadoxine has not been achieved in Choco. MMGs found matched the clonal lineage E variant 1 previously reported in northwestern Colombia.

**Supplementary Information:**

The online version contains supplementary material available at 10.1186/s12936-022-04328-x.

## Background

Malaria transmission in the Americas is characterized as unstable, heterogeneous, and predominantly of low intensity [1]. In addition, a downward trend of malaria cases was observed between 2000 and 2014, which changed in 2015, when a significant resurgence of epidemic transmission in Venezuela led to increase in number of cases along with increased transmission in endemic areas of Brazil, Colombia, Guyana, Nicaragua, and Panama; as well as outbreaks in countries that were moving towards elimination [2]. In 2019, more than 86% of malaria cases in the Americas, were concentrated in Venezuela, Brazil, and Colombia [3].

Colombia is endemic for *Plasmodium falciparum* and *Plasmodium vivax* and approximately 70,000 malaria cases are reported annually by the public health services, mainly in departments of Choco, and Nariño [4]. Despite the relatively large number of malaria cases reported each year and the use of different anti-malarial treatments in recent decades, little is known about the molecular markers of drug resistance and genetic diversity of *P. falciparum* in the country. Since 2010, the first-line regimen for treating uncomplicated *P. falciparum* malaria in Colombia has been artemether–lumefantrine (AL). Prior to the use of this artemisinin-based combination therapy (ACT), chloroquine (CQ), sulfadoxine-pyrimethamine (SP), and amodiaquine (AQ) were widely used, until high rates of therapeutic failure were reached [5–7]. CQ monotherapy was employed until the early 1980s, but when resistance to this anti-malarial emerged, SP in combination with CQ or AQ became the therapeutic regimens utilized. In 1999, AQ-SP was implemented as the first-line treatment for uncomplicated *P. falciparum* malaria and from 2006, two artemisinin-based combinations, artesunate-mefloquine and AL, were introduced.

*Plasmodium falciparum* resistance to CQ and SP has been associated with point mutations in the chloroquine-resistance transporter (*pfcrt* gene) [8], and the dihydrofolate reductase (*pfdhfr*) and dihydropteroate synthase (*pfdhps*) genes [9], respectively. The *pfcrt* point mutation K76**T** is critical to confering CQ resistance, but mutations in other codons are also associated with resistance [8]. Resistance to sulfadoxine-pyrimethamine is associated with the accumulation of mutations at codons 50, 51, 59, 108, and 164 in the *pfdhfr* gene (codon 50 being more relevant in South America) and 436, 437, 540, and 581 of the *pfdhps* gene, respectively [9].

In 2018, a therapeutic efficacy study (TES) was carried out to evaluate the efficacy of AL for the treatment of uncomplicated *P. falciparum* malaria in Quibdo, Choco, together with the evaluation of the confirmed molecular marker of resistance to artemisinin in the *pfk13* gene*,* and to unconfirmed marker for lumefantrine resistance, *pfmdr1* gene [10]. Although TESs are indispensable to assess the efficacy of anti-malarial drugs, molecular surveillance is helpful to detect the emergence of mutations associated with drug resistance and signal recovering sensitivity to an anti-malarial used in the past. To evaluate if the prevalence of wild-type genotypes of *pfcrt, pfdhfr, and pfdhps* genes has changed over time since their withdrawal in Colombia, molecular testing of samples collected during the 2018 TES was conducted. In addition, the genetic diversity of infections using seven neutral microsatellites (NMSs) loci was assessed.

## Methods

### Ethics statement

Samples for this study were obtained from a TES conducted in Quibdo in 2018 [10]; as part of the TES, written informed consent for secondary use of remnant samples were obtained. The TES protocol was reviewed and approved by the Institutional Ethics Committee of the Colombian National Institute of Health (Protocol CEMIN 2-2018, Minute #5 of March 22, 2018) and the PAHO Ethics Review Committee (PAHOERC) (PAHO 2018-04-0029). CDC considered the study a public health evaluation (reference number: 2018-063).

### Study site, sample collection, DNA extraction, and PET-PCR

Quibdo is the capital of Choco department, located in northwestern Colombia (lat. 5° 41′ 32″ N, long. 76° 39′ 29″ W) on the banks of the Atrato river in the Pacific region, at an altitude of 43 m above sea level, the average temperature is 28 °C and annual humidity oscillates between 86 and 88%. The Pacific region is separated from Amazon and Orinoco region by the Andes Mountain range. Quibdo has a population of 116,087, mainly consisting of Afro Colombians (87.5%), of which 65% is settled in the urban area. Quibdo contributes 6 to 11% of the total cases of malaria reported in Colombia.

Samples collected at patient enrollment in the TES were used in this study. Parasite DNA was extracted from dried blood spots collected on filter paper using the QIAamp® DNA Micro Kit (Qiagen, Hilden, Germany) following the manufacturer’s instructions. The *Plasmodium* species was confirmed by a real-time PCR, photo-induced electron transfer polymerase chain reaction (PET-PCR) as previously described [11].

### Sanger sequencing for molecular markers of resistance

*Plasmodium falciparum* samples were genotyped using PCR or nested PCR followed by Sanger sequencing to evaluate mutations at *pfcrt* gene (codons 72–76), *pfdhfr* gene (codons 51, 59, 108, and 164), and *pfdhps* gene (codons 436, 437, 540, and 581). Primer sequences and PCR cycling conditions for each gene are reported elsewhere [12–14]. Briefly, primary PCR was performed with 1 μL genomic DNA, 7.5 μL of 2X master mix (Promega, Madison, WI, USA), and 0.27 μM each primer in 15 μL final volume for *pfdhfr* and *dhps* genes. Conditions for *pfcrt* gene were the same, except 1.2 μL of DNA and a final concentration of 0.33 μM each primer. Nested PCR was done for *pfdhps* and *pfcrt* genes. PCR products were purified with ExoSAP-it reagent (New England Biolabs, Ipswich, MA, USA) and sequenced using the Sanger method (Genewiz INC, NJ, USA). Laboratory *P. falciparum* strains, 3D7 and 7G8, were used as wild-type and mutant-type controls, respectively. Sequence analysis was performed using Geneious® 7.1.7 software (Biomatters, Auckland, New Zealand) using the *P. falciparum* 3D7 reference strain for *pfcrt* (PF3D7_0709000), *pfdhfr* (PF3D7_0417200), and *pfdhps* (PF3D7_0810800) genes. Mutant alleles were identified, and the frequency of each allele was determined.

### Neutral microsatellites

All samples were genotyped using a set of seven NMSs to identify polyclonal infections. The microsatellite loci amplified were on chromosome (chr) 2 (C2M34), chr 3 (C3M69), chr 4 (Polyα), chr 6 (TA1 and TA109), chr 10 (2490), and chr 12 (PfPK2) [15–17]. Amplification products labeled fluorescently with FAM or HEX of the 7 loci were followed by fragment electrophoresis on a capillary sequencer ABI 3130 xl Genetic Analyzer (Applied Biosystems, Foster city, CA, USA) and then fragment sizes were scored using GeneMapper®V2.7.0 software (Applied Biosystems, Foster city, CA, USA). PCR cycling conditions for each locus [15–17] are registered elsewhere. Samples that carried a single allele at each locus were considered monoclonal infections and, when more than one allele was found at any ≥ 1 locus, they were considered polyclonal infections. Presence of alleles (multiple peaks) was evaluated by locus and minor alleles were scored if they were more than 33% the height of the peaks corresponding to the predominant alleles. Genetic variation at each locus was quantified by the allelic diversity, which was calculated in terms of expected heterozygosity (*H*_*e*_) using the formula ${H}_{e}=\left[\frac{n}{n-1}\right]\left[1-\sum {p}_{i}^{2}\right]$, where n is the number of *P. falciparum* isolates genotyped for a particular locus and p_*i*_ is the frequency of the _*i*_th allele. The sampling variance of *H*_*e*_ was estimated as $\left[\frac{2\left(n-1\right)}{{n}^{3}}\right]\left(2\left(n-2\right)\left[\sum {p}_{i}^{3}-{\left(\sum {p}_{i}^{2}\right)}^{2}\right]\right)$ [18]. Infections containing more than one allele at any of the seven NMSs were not included in the analysis.

## Results

### Molecular markers of resistance

A total of 88 samples were processed of which 85 could be confirmed as *P. falciparum* by PET-PCR. Among these 85 samples, sequencing of the *pfcrt, pfdhfr,* and *pfdhps* genes was successful in 72 (84.7%), 79 (92.9%), and 73 (85.9%) samples, respectively. For the *pfcrt* gene, all isolates carried the K76**T** and N75**E** mutant alleles. Only two mutant alleles, N51**I** and S108**N,** were observed in the *pfdhfr* gene, and for the *pfdhps* gene wild-type alleles prevailed in all codons investigated except in codon 437, where 32 (43.8%) isolates carried the mutant allele A437**G** (Table 1).

**Table 1:** Observed *P. falciparum pfcrt, pfdhfr,* and *pfdhps* resistance alleles, Quibdo, Colombia, 2018

Gene	Polymorphic positions	Genotype	Genotype frequency*	
			n	(%)
*pfcrt*	C72**S**/V73/M74**I**/N75**E**/K76**T**	CVM**ET**	72/72	100.0
*pfdhfr*	N51**I**/C59**R**/S108**N**/I164**L**	**I**C**N**I	79/79	100.0
*pfdhps*	A437**G**/K540**A**/A581**G**	AKA	41/73	57.5
*pfdhps*	A437**G**/K540**A**/A581**G**	**G**KA	32/73	43.8

^*^The frequency was calculated only with the samples that amplified

In bold are mutant-type alleles

### Genetic diversity

A complete NMS profile was obtained for 58.8% (50/85) of the samples, 22.4% (19/85) lacked allele amplification in one locus and 18.8% (16/85) in ≥ 2 loci. Monoclonal infections were predominant (96%) and only 2 samples showed evidence of polyclonal infections. Fifteen alleles were found and of these, one had not been previously reported in Colombia [19–21]; allele size 193 bp in TA109 was found in a polyclonal infection and was different from those previously reported in Choco. Mean number of alleles per locus (2.0 ± 0.57) and mean *H*_*e*_ (0.17 ± 0.03) were low (Table 2).

**Table 2:** Allelic diversity in seven NMS loci and expected heterozygosity from isolates collected in Quibdo, Choco

NMS	TA1	POLYα	PfPK2	TA109	2490	C2M34	C3M69	Mean No. of allele (± s.d)	Mean (± s.d)
Tandem repeats	ATT	ATT	TAA	AAT	TAA	AT	TA		
No. of detected alleles	1	2	3	1	3	2	2	2.0 (0.57)	
*He	0	0.04	0.33	0	0.19	0.45	0.16		0.17 (0.03)

*He: heterozygosity

n = 48, monoclonal infections

**Fig. 1:**
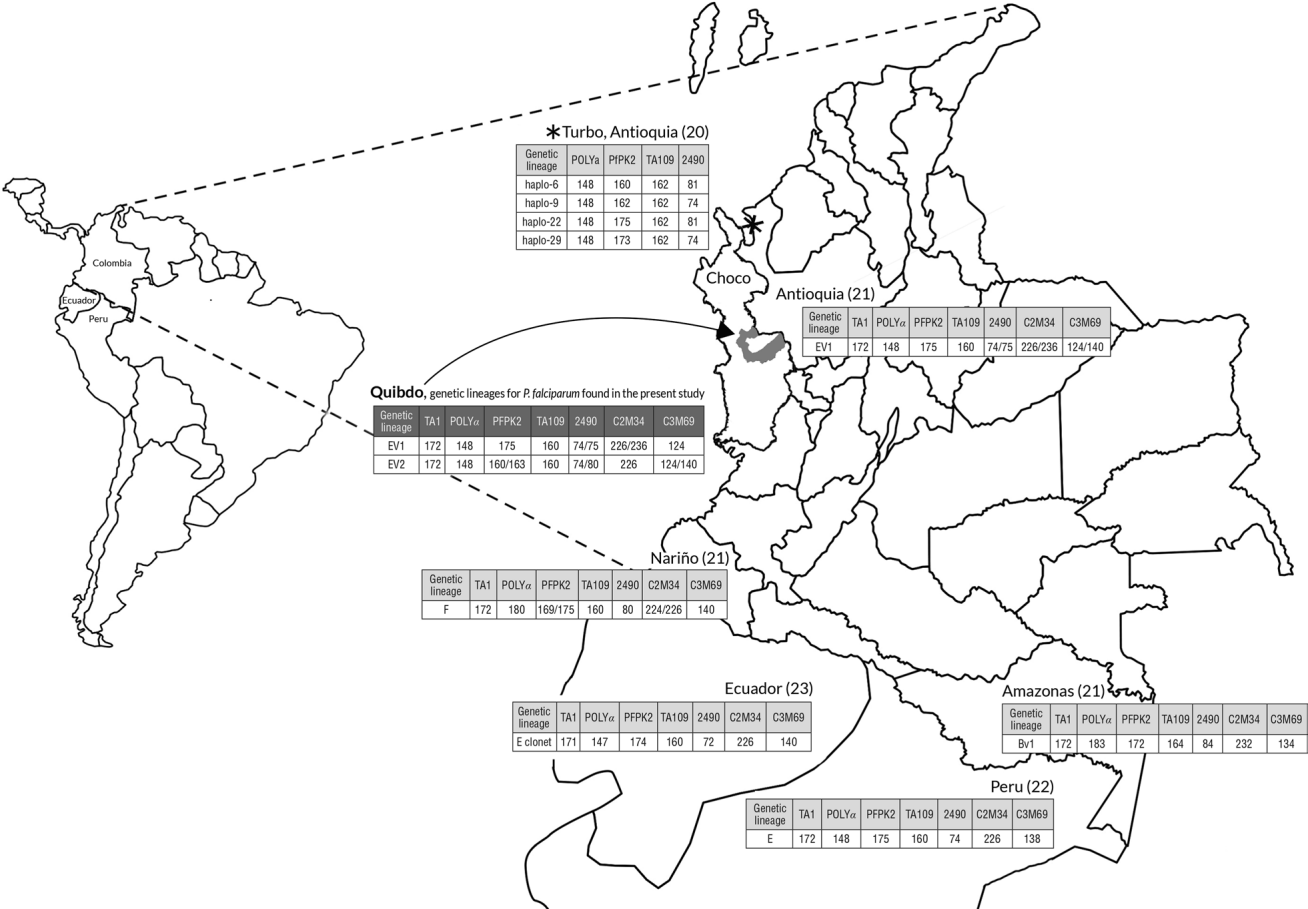
Variants of *P. falciparum* genetic lineage E found in Quibdo, Choco, 2018. Map of Colombia showing *P. falciparum* genetic lineages E (Ev1 and Ev2) found in Quibdo, Choco and genetic lineages found in other departments of Colombia and in neighbouring countries. Turbo in Antioquia (20), Antioquia (21), Nariño (21), Amazonas (21), Perú (22), and Ecuador (23)

NMS multilocus genotypes (MMGs) were built from the samples with monoclonal infection by combining the results of the seven microsatellites and coded with numbers. Nine MMGs were identified from samples with the complete profile (Table 3) revealing the existence of related multilocus genotypes. MMG 1 and MMG 2 were the most prevalent genotypes and between each MMG there were minor variations in NMS composition, for example, MMG 1 and MMG 2 shared a similar genetic profile differing only in one allele size found in C2M34 (226 vs. 236 bp). The remaining seven MMGs were found in one or three samples and were related to MMG 1, either by sharing some of the alleles or by their close allele sizes. The allele sizes detected in 37 (77.1%) isolates (MMGs 1, 2, and 3) matched the clonal lineage E variant 1 (E_v1_) previously reported [21], which exhibits the following profile TA1: 172, Polyα: 148, PfPK2: 175, TA109: 160, 2490: 74/75, C2M34: 236/226, C3M69: 124. E_v1_ is a variant of the clonal lineage E, which was described earlier in Peru [22] and Ecuador [23]. Eleven (22.9%) isolates had different alleles from those described for E_v1_ such as 154 in Poly-α, 160 and 163 in PfPK2, and 80 in 2490. This finding suggests that in Quibdo another variant of the clonal line E circulates, which was named variant 2 (E_v2_). This variant was found in 8 isolates (MMGs 5, 6, 7, and 8) and its microsatellite profile was TA1: 172, Polyα: 148, PfPK2: 160/163, TA109: 160, 2490: 74/80, C2M34: 226, and C3M69: 124/140 (Fig. 1).

**Table 3:** Neutral microsatellites multilocus genotypes (MMG) in Quibdo, Colombia (n = 48), 2018

Neutral microsatellite multilocus genotypes (MMG)								
MMG	Frequency n (%)	Allele size (bp)						
		TA1	Polyα	PfPK2	TA109	2490	C2M34	C3M69
1	20 (41.7)	172	148	175	160	74	226	124
2	16 (33.3)	172	148	175	160	74	236	124
3	1 (2.1)	172	148	175	160	75	226	124
4	2 (4.2)	172	148	175	160	80	226	124
5	1 (2.1)	172	148	160	160	74	226	140
6	2 (4.2)	172	148	160	160	80	226	124
7	3 (6.2)	172	148	163	160	74	226	124
8	2 (4.2)	172	148	163	160	74	226	140
9	1 (2.1)	172	154	160	160	74	226	140

n: number of single clone isolates

No association was observed between the two resistance genotypes (combination of *pfcrt*, *pfdhfr,* and *pfdhps* resistance alleles) and MMGs found in this study; however, parasites with MMGs 2, 3, and 8 carried the CVM**ET**/**I**C**N**I/AKA genotype and parasites with MMGs 4, 7, and 9 harbored CVM**ET**/**I**C**N**I/**G**KA. Parasites with MMGs 1 and 6 showed both genotypes (see Additional file 1). When comparing the isolates belonging to E_v1_ with the rest of the isolates found in Quibdo, samples shared the same drug resistance genotypes CVM**ET**/**I**C**N**I/AKA or CVM**ET**/**I**C**N**I/**G**KA, being more common the genotype CVM**ET**/**I**C**N**I/AKA within the variant E_v1_ (68,8%). Considering that the samples analysed in a previous report [10] are the same as in this study, *Pfmdr1* genotypes N**F**SDD/N**F**SD**Y** previously reported [10] were found in both the E_v1_ and the remaining isolates; however, genotype N**F**SDD was detected mostly in E_v1_ (25%) and only in isolates with an allele size of 226 bp in C2M34 (see Additional file 1).

## Discussion

The monitoring of molecular markers of resistance is useful for detecting changes in the parasite genome associated with anti-malarial drugs resistance over time. In this study, only one CQ-resistant genotype (CVM**ET)** was found, in contrast to the three genotypes (CVM**ET**/CVMN**T**/CVI**ET**) previously reported in samples from the Colombian Pacific [24]. Field isolates carried mutant K76**T** in *pfcrt* gene, indicating that, despite the withdrawal of CQ from national treatment guidelines for *P. falciparum* in 1998, resurgence and re-expansion of wild-type *pfcrt P. falciparum* has not occurred. A similar situation was reported in a previous study carried out in three Colombian malaria-endemic areas [25] and other countries in the Americas [26, 27]. This finding contrasts with what was observed in some countries of Africa, like Malawi [28, 29], where the recovery of CQ-sensitive wild-type *pfcrt P. falciparum* occurred in as little as 10 years after the use of CQ was discontinued.

As a result of CQ resistance in Colombia, SP was adopted in 1981; high and moderate levels of therapeutic failure were quickly observed in the Amazon basin [7] and northwestern regions [6], respectively. The analysis of molecular markers of resistance to SP in this study shows a predominance of double mutant (**I**C**N**I) for *pfdhfr,* while mutations in the *pfdhps* gene were only observed at codon 437 (43.8%), an indication that full resistance to sulfadoxine was not achieved in circulating parasites in Choco*.* In addition, ACT replaced the combined therapy SP with AQ for uncomplicated *P. falciparum* malaria in 2008, and since then there has been no selective pressure with SP. Similar resistance profiles were observed in northwest Colombia in previous studies [19, 30, 31]. This likely slow emergence of mutations in *pfdhps* in parasites from Quibdo could be explained, in part, by the use of multiple drug regimens (SP in combination with CQ or AQ plus primaquine as a gametocytocidal agent) unlike Brazil and the Peruvian Amazon region, where SP monotherapy was used and parasites with double and triple mutations predominate.

The frequency of mutations associated with resistance to CQ (K76**T** 100%) [25, 32, 33] and SP (N51**I**/S108**N** 93–100% and A437**G** 65–70%) [19, 30, 31] has remained constant in northwest Colombia for at least 10 years since introduction of ACT, suggesting the fixation of resistant alleles [12, 34] in Choco. This situation could be explained by the strong selective pressure exerted by the drugs that fixed the mutant allele in this population, considering that the frequency of these mutant alleles is 100% and it has prevailed unchanged over the last decade in this part of the country. Other possible reasons for fixation of these mutant alleles include CQ and SP drug pressure not allowing for expansion of sensitive strains. Although CQ and SP are no longer used to treat *P. falciparum* malaria in Colombia, CQ constitutes the first-line regimen for vivax malaria and self-medication with CQ for non-laboratory confirm malaria has also been observed in 32% of symptomatic individuals [35]. In addition, the prevalence of CVM**ET** over time may be because this haplotype, like **S**VMN**T** found in Papua New Guinea and Brazil, has no fitness disadvantage over the sensitive one even in the absence of drug pressure [36]. Finally, it is likely that no wild-type populations, able to replace the resistant populations, were left in Colombia after the development of resistance.

When comparing the number of alleles per microsatellite locus against collected samples in Quibdo during 2001–2007 [19] and in Turbo during 2002–2008 [20], a town located in Antioquia department in northwestern Colombia, more alleles were detected at that time (six and seven for Polyα and six and nine for PfPK2). Higher diversity values (expected heterozygosities) were found in both studies, 0.915 for the first, and 0.35 to 0.18 for the second, compared to 0.17 in the present study. These results points to a progressive reduction in the level of genetic diversity in *P. falciparum* from Quibdo, as well as a decrease in the frequency of multiple infections. These findings suggest the circulating *P. falciparum* population have a more clonal composition because these populations have undergone bottlenecks possibly caused by actions of national malaria control programme or interventions focused on improving access to timely diagnosis and adequate treatment, and the coverage in the use of insecticide-treated bed nets such as World Fund Malaria Project implemented in Colombia between 2010 and 2015. Additionally, alleles 172 bp in TA1 and 160 bp in TA109 seem to be fixed in parasites from Quibdo.

Of the 48 isolates analysed for NMS, 77.1% shared the same microsatellite profile of the clonal lineage E_V1_, which was described in the neighbouring department of Antioquia [21], and 16.7% showed a new variant of the clonal lineage E, named E_v2_ (Fig. 1). This variant is characterized by having a genetically identical set of markers, but variable at others, with common ancestors occurring more than 15 years ago, according to allele sizes reported in a study carried out in Quibdo with samples collected in 2001 and 2006 [19].

When comparing the haplotypes found in Quibdo with other haplotypes reported in Colombia, it is evident that the clonal lineage E variant 1 predominates in this town and it is characteristic in this area of the country; this same variant was reported in samples from Antioquia collected in 2012, indicating that in the northwestern zone of Colombia there is a predominance of variants derived from genetic lineage E. The genetic lineage Bv1 predominates in the Amazonas department 21, variant previously observed in Peru 22, and the genetic lineage F 21 in Nariño department (southwestern Colombia) (Fig. 1). The foregoing allows us to suggest that specific genetic lineages predominate in the different endemic areas of malaria in Colombia.

## Conclusions

Overall, the frequency of anti-malarial drug resistance genotypes and NMS multilocus genotypes was studied in northwestern Colombia to explore possible genomic adaptations in the parasite in response to changes in the anti-malarial policy and genetically characterize *P. falciparum* populations. Monitoring of molecular markers of anti-malarial drugs resistance should be considered as part of regular in vivo efficacy trials in malaria-endemic countries to timely detect emergence of polymorphisms conferring decreased susceptibility to anti-malarials, especially since self-medication, the use of subtherapeutic doses, gold mining activities associated with malaria outbreaks, and human mobility and migration are common in Colombia.

## Supplementary Information

**Additional file 1: Table S1.** Alleles found in samples from Quibdo, 2018. *Pfmdr1 data is already published (10). Tabular data with the alleles found in *crt, dhfr, dphs*, and *mdr1* genes and 7 neutral microsatellites in 42 samples from Quibdo, Colombia, 2018. Red letter indicates a mutant allele. N/A indicates no allele amplification at that locus.

## Data Availability

Database is available in supplementary information.

## Abbreviations

ACT: Artemisinin-based combination therapy
AL: Artemether–lumefantrine
AQ: Amodiaquine
CQ: Chloroquine
DNA: Deoxyribonucleic acid
He: Heterozygosity
MMG: NMS multilocus genotypes
NMS: Neutral microsatellites
PCR: Polymerase chain reaction
PET-PCR: Photo-induced electron transfer-PCR
SP: Sulfadoxine-pyrimethamine
TES: Therapeutic efficacy study
